# Modulation of inhibitory activity of xylanase - α-amylase inhibitor protein (XAIP): binding studies and crystal structure determination of XAIP- II from *Scadoxus multiflorus *at 1.2 Å resolution

**DOI:** 10.1186/1472-6807-10-41

**Published:** 2010-11-20

**Authors:** Sanjit Kumar, Nagendra Singh, Biswajit Mishra, Divya Dube, Mau Sinha, S Baskar Singh, Sharmistha Dey, Punit Kaur, Sujata Sharma, Tej P Singh

**Affiliations:** 1 Department of Biophysics, All India Institute of Medical Sciences, New Delhi, India

## Abstract

**Background:**

Plants produce a wide range of proteinaceous inhibitors to protect themselves against hydrolytic enzymes. Recently a novel protein XAIP belonging to a new sub-family (GH18C) was reported to inhibit two structurally unrelated enzymes xylanase GH11 and α-amylase GH13. It was shown to inhibit xylanase GH11 with greater potency than that of α-amylase GH13. A new form of XAIP (XAIP-II) that inhibits α-amylase GH13 with a greater potency than that of XAIP and xylanase GH11 with a lower potency than that of XAIP, has been identified in the extracts of underground bulbs of *Scadoxus multiflorus*. This kind of occurrence of isoforms of inhibitor proteins is a rare observation and offers new opportunities for understanding the principles of protein engineering by nature.

**Results:**

In order to determine the structural basis of the enhanced potency of XAIP-II against α-amylase GH13 and its reduced potency against xylanase GH11 as compared to that of XAIP, we have purified XAIP-II to homogeneity and obtained its complete amino acid sequence using cloning procedure. It has been crystallized with 0.1 M ammonium sulphate as the precipitating agent and the three-dimensional structure has been determined at 1.2 Å resolution. The binding studies of XAIP-II with xylanase GH11 and α-amylase GH13 have been carried out with surface plasmon resonance (SPR).

**Conclusion:**

The structure determination revealed that XAIP-II adopts the well known TIM barrel fold. The xylanase GH11 binding site in XAIP-II is formed mainly with loop α3-β3 (residues, 102 - 118) which has acquired a stereochemically less favorable conformation for binding to xylanase GH11 because of the addition of an extra residue, Ala105 and due to replacements of two important residues, His106 and Asn109 by Thr107 and Ser110. On the other hand, the α-amylase binding site, which consists of α-helices α6 (residues, 193 - 206), α7 (residues, 230 - 243) and loop β6-α6 (residues, 180 - 192) adopts a stereochemically more favorable conformation due to replacements of residues, Ser190, Gly191 and Glu194 by Ala191, Ser192 and Ser195 respectively in α-helix α6, Glu231 and His236 by Thr232 and Ser237 respectively in α-helix α7. As a result, XAIP-II binds to xylanase GH11 less favorably while it interacts more strongly with α-amylase GH13 as compared to XAIP. These observations correlate well with the values of 4.2 × 10^-6 ^M and 3.4 × 10^-8 ^M for the dissociation constants of XAIP-II with xylanase GH11 and α-amylase GH13 respectively and those of 4.5 × 10^-7 ^M and 3.6 × 10^-6 ^M of XAIP with xylanase GH11 and α-amylase GH13 respectively.

## Background

Plants produce a wide range of proteinaceous inhibitors that protect them from the unwanted hydrolytic effects of endogenous enzymes as well as from those of infecting micro-organisms. Recently, a new inhibitor protein with two independent binding sites designated as XAIP (Xylanase and α-amylase inhibitor protein) was isolated from *Scadoxus multiflorus *[1]. This protein showed sequence homologies of 48% with heavamine, another plant protein with chitinase activity [2], 39% with concanavalin (con-B) [3] and 11% with narbonin [4]. The latter two did not act as chitinases while their precise functions are still unkonown. XAIP also showed a 36% sequence homology with XIP-I (xylanase inhibiting protein) that inhibits xylanases GH10 and GH11. It also lacks chitinase-like activity [5,6]. Structurally, they all adopt (β/α)_8 _barrel fold. Because of an extra α-helix α8' in the structures of these proteins, they all are classified into a sub-family of glycosyl hydrolyses 18C (GH18C) as a part of the larger family of GH18 proteins that consists of mainly chitinases [7] and various other proteins of unknown functions [3,4,8]. The proteins of sub-family GH18C show significant sequence variations while they adopt an overall similar scafolding. These proteins differ greatly in their functional specificities [9,10]. We report here a new form of XAIP (XAIP-II) which inhibits xylanase GH11 with a reduced potency whereas it binds to α-amylase with a considerably enhanced binding affinity as compared to XAIP [1]. The two forms, XAIP-II and XAIP show a sequence homology of 87% while 13% sequence variations occur mostly in the regions of ligand binding sites. The detailed structure determination of XAIP-II has allowed us to examine the reasons for the lack of chitinase activity, loss of carbohydrate binding capability, reduction in xylanase specific activity and significant increase in the potency of α-amylase inhibition.

## Results and Discussion

### Sequence analysis

**Figure 1:**
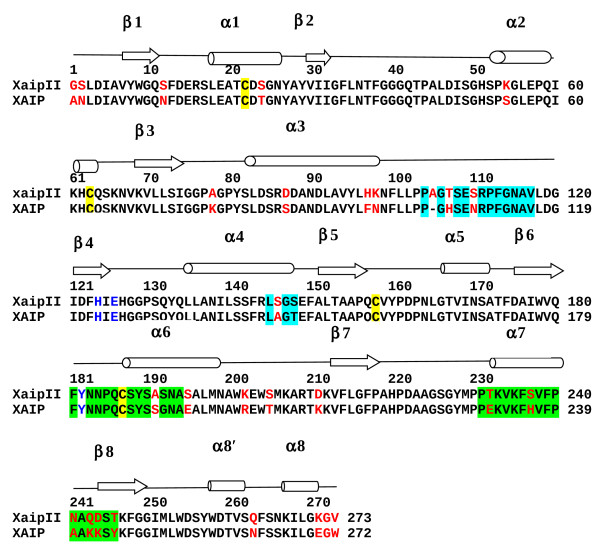
**Sequence alignment of XAIP-II (present structure) and XAIP **[1]. The secondary structure elements i.e. α-helices (α1 to α8'/α8) and β-strands (β1 to β8) are represented by cylinders and arrows respectively. The regions of polypeptide chain involved in the binding site with xylanase GH11 are highlighted with cyan background and those with α-amylase GH13 are highlighted with green background. The amino acids corresponding to the chitinase active site are indicated in blue colour while cysteine residues are shaded in yellow. The differences in the sequences of XAIP and XAIP-II are indicated in red.

The amino acid sequence of XAIP-II shows a sequence homology of 87% with that of XAIP (Figure 1). XAIP-II consists of 273 amino acid residues (accession number: HM474410). The amino acid residue at position 77 (in the numbering scheme of XAIP-II) in generally different in XAIP-like proteins indicating an important structural and functional role of this residue although it is same in the sequences of XAIP-II and XAIP. Interestingly, a neighbouring residue at position 78 is quite different in the two forms as it is alanine in XAIP-II whereas it is lysine in XAIP [1]. The difference in the size of the side chains of two residues suggest that it may have significant local influence on the structure. The protein chain of XAIP-II is longer than that of XAIP by one amino acid residue as Ala105 is extra in XAIP-II. This is part of an important loop, Pro103 - Phe113 which is located between α-helix α3 and β-strand β4. In the same loop, residues His106 and Asn110 of XAIP have been replaced by residues Thr107 and Ser110 in XAIP-II. The residues Ser190, Gly191, Glu194, Arg201, Thr204, Lys210, Glu231 and His236 (in the α-amylase binding site consisting of α-helices α6, α7 and loop (β6-α6) have been replaced by Ala191, Ser192, Ser195, Lys202, Ser205, Asp211, Thr232 and Ser237 in XAIP-II. The sequence of XAIP-II shows an identity of 48% with the sequence of hevamine which is plant protein with chitinase activity. Hevamine also belongs to glycosyl hydrolase (GH) family 18C and has the characteristic combination of residues Asp125, Glu127 and Tyr183 for chitinase activity whereas the corresponding residues in XAIP-II are His124, Glu126 and Tyr182 indicating a loss of chitinase activity. It may be noted that the sequence variations between XAIP and XAIP-II are generally confined to the regions of binding sites (Figure 1).

### Determination of K_D _by surface plasmon resonance

**Figure 2:**
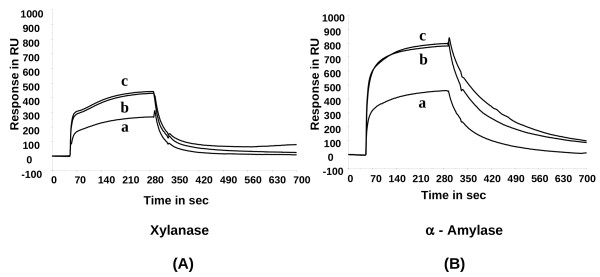
**The SPR-sensograms for the bindings of XAIP-II with (A) xylanase GH11 and (B) α-amylase GH13**. XAIP-II was immobilized on the chip and the increasing concentrations (1.8 μM, 3.6 μM and 5.4 μM) of enzymes xylanase GH11 and α-amylase GH13 were used in the mobile phase in separate experiments corresponding to curves a. b and c.

The molecular interactions between XAIP-II and xylanase GH11 and α-amylase GH13 were studied in the real time using a biosensor based surface plasmon resonance (SPR) [11]. The inhibitor protein XAIP-II was immobilized as a ligand on the dextran surface of a chip CM5 whereas the enzymes *Penicillium funiculosum *xylanase GH11 and *Bacillus licheniformis *α-amylase GH13 were used as the analytes over the surface. The sensograms for the interactions of XAIP-II with xylanase GH11 and α-amylase GH13 are shown in Figures 2A and 2B respectively. The increase of resonance unit (RU) from the base lines represents the binding of xylanase GH11 and α-amylase GH13 to the immobilized inhibitor protein. The plateau line represents the steady state equilibrium phase of the interactions between the inhibitor and the enzymes whereas the decrease in RU from the plateau represents dissociation phase. As seen from Figures 2A and 2B, the dissociation phase is slower in the case of α-amylase (Figure 2B) than that of xylanase (Figure 2A) indicating a stronger interaction between XAIP-II and α-amylase GH13 (3.4 × 10^-8 ^M) than that of XAIP-II and xylanase GH11 (4.2 × 10^-6 ^M) as estimated using BIA evaluation software [12]. This is in contrast to the earlier reports [1] where the interaction of XAIP and α-amylase GH13 (3.6 × 10^-6 ^M) was weaker than that of XAIP and xylanase GH11 (4.5 × 10^-7 ^M). These SPR data clearly show that XAIP-II like that of XAIP inhibits both xylanase GH11 and α-amylase GH13 but the affinities of bindings have reversed indicating the significance of variations in the sequences of XAIP-II and XAIP.

### Quality of the model

At the end of the refinement, the values of R_cryst _and R_free _factors were 15.8% and 18.6% respectively. The overall geometry of the XAIP-II structure refined to 1.2Å resolution was good with a molprobity [13] score of 85 percentile. The average thermal B-factor was 15.8Å^2 ^while the 92.3% residues were found in the most favored regions of the Ramachandran plot [14] as indicated by PROCHECK [15].

### Overall structure of XAIP-II

**Figure 3:**
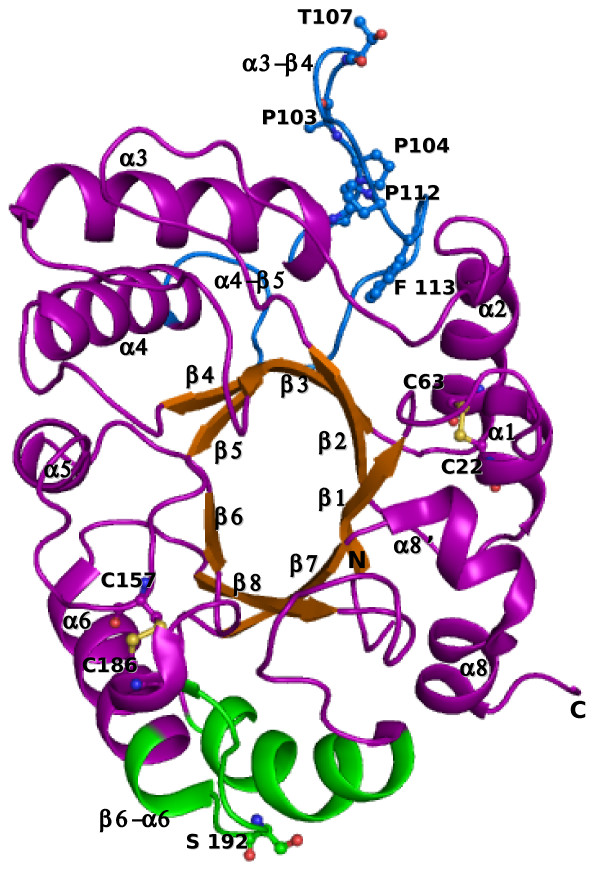
**The schematic representations of the structure of XAIP-II**. The α-helices (purple) and β-strands (orange) are labelled from 1 to 8. Two disulfide bonds are indicated in yellow. The loop α3-β4 (102-118) and α4-β5 (146-149) form the surface which is involved in the binding with xylanase GH11 and are shown in blue. Some important residues are shown in ball and stick model. The loop β6-α6 (180-193), α-helix α6 (1932-06), loop β7-α7 (2182-30) and α-helix α7 (230-243) from the opposite surface of the protein which are assumed to be involved in the binding with α-amylase are indicated in magenta. The residues are shown in ball and stick representation. The figure was drawn using PyMol [26].

The polypeptide chain of XAIP-II adopts the triosephosphate isomerase (TIM) barrel folding with an eight stranded β-sheet that forms an inner circle while the nine α-helices constitute the outer loop (Figure 3). Because of the presence of an additional α-helix α8' (residues, 255-261) this protein is grouped in the subclass of family 18C proteins (GH18C) of the glycosyl hydrolases (GH 18) family. There are two non-proline cis peptide bonds, Gly33-Phe34 and Trp254-Asp255 in the structure while one proline cis peptide bond, Tyr160-Pro161 has also been observed. The non-proline cis peptides belong to loops β2-α2 and β8-α8' respectively while the proline cis peptide is located in the β5-α5 loop. When the two structures of XAIP-II and XAIP were superimposed on each other, the r.m.s. shift was found to be 1.5Å. However, the superimposition of C^α ^traces of XAIP-II and XAIP shows an r.m.s shift of only 0.7Å indicating that the polypeptide chains of two proteins show an overall similar conformations. However, the regions comprising helices, α2, α3, α7, β-strands, β2, β5, β6 and loops β2-α2, α3-β4, β5-α5 and α6-β6 show significant r.m.s shifts in their Cα postions.

### Comparisons of the structures of XAIP-II and XAIP

Both molecules lack chitinolytic activity. Both structures differ significantly in the regions of two interaction sites corresponding to xylanase GH11 binding site and α-amylase GH13 binding site. The structures show that the carbohydrate binding channel is relatively less obstructed in XAIP-II than that in XAIP. The binding constant of XAIP-II with xylanase GH11 is less than that of XAIP while the binding affinity with α-amylase GH13 is considerably enhanced.

### Carbohydrate binding channel

**Figure 4:**
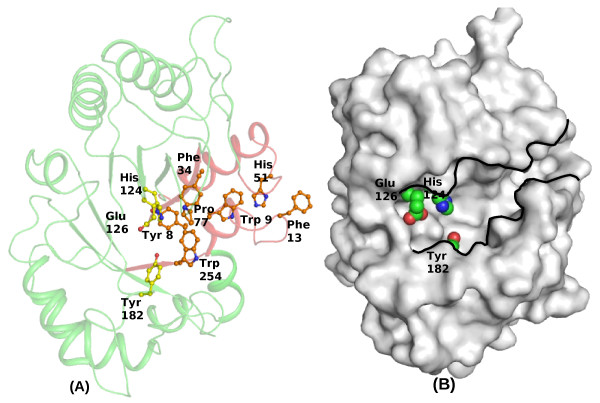
**The carbohydrate binding channel in XAIP-II**. (A) The backbone tracing of XAIP-II with carbohydrate binding channel in red are illustrated. The important residues that obstruct the channel are also shown. The residues corresponding to active side residues of hevamine have been shown in yellow. (B) The molecular surface drawn using GRASP [27] is shown with carbohydrate binding-channel. The three active site residues are indicated as space filling models.

Both XAIP-II and XAIP maintain impaired carbohydrate-binding channels. The so called carbohydrate-binding site is formed with segments Ala45-Ser52, Ala6-Phe13, Leu56-Lys66, Gly76-Gly79, Met252-Tyr257 and Cys22-Ala28 (Figure 4). The prominent residues that determine the inner shape of the channel are Phe13, Asp47, His49, Pro77, Ala78, Trp254 and Asp256. The corresponding residues in the catalytically active hevamine are Gly, Asn, Ala, Gly, Ile, Trp and Ser respectively. The presence of Phe13 at the entrance of the channel in XAIP-II obstructs the entry of carbohydrates to the binding site. It may be noted that Phe13 is one of the corner residues of a tight type I' β-turn conformation and the position of its side chain is locked at the observed site. On the other hand, the presence of inwardly protruding Pro77 in the middle of the inner β-barrel blocks the channel passage right in the middle just before the positions of active site residues. As a result, the internal space of the channel is considerably smaller than that of chitinases [7]. As far as the differences between the impaired carbohydrate channels of XAIP-II and XAIP is concerned, the most notable variation is observed in the proximity of chitin binding site where Ala78 in XAIP-II has replaced Lys78 of XAIP. The structure analysis shows that the side chain of Lys78 interacts with Asn36, Asp47 and Ser49 as well as with several solvent water molecules. On the other hand, Ala78 in XAIP-II is oriented outwardly from the channel and hence does not exert any effect on the carbohydrate binding channel. Therefore, one of the internal surfaces of the hydrophobic channel is relatively more rigid in XAIP as compared to that of XAIP-II. In spite of repeated attempts of co-crystallizing XAIP-II with various chitin fragments including N-acetyl glucosamine monomer and other model sugar molecules, we did not succeed in getting crystals of the complex of XAIP-II with sugar. The soaking experiment also did not yield the desired crystals of the complex of XAIP-II with sugars indicating that the carbohydrate binding channel is not optimally formed in XAIP-II.

### Binding with xylanase GH11

**Figure 5:**
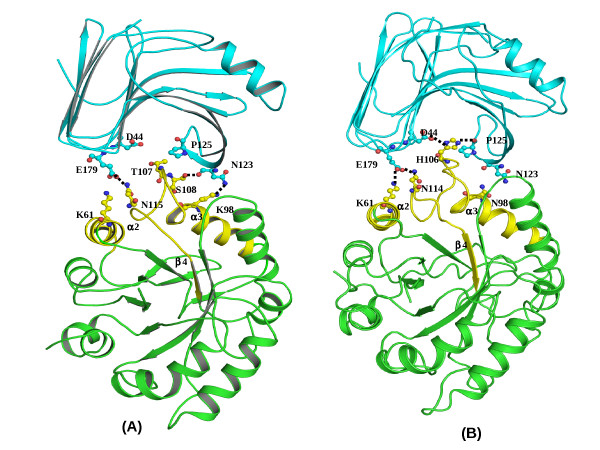
**The comparisons of interactions between xylanase GH11 (blue) with (A) XAIP-II (green) and (B) XAIP (green)**. These were modeled using docking methods [16,17] are shown. The hydrogen bonds are indicated by dotted lines. The binding regions of both XAIP-II and XAIP are shown in yellow colour.

In XAIP-II, the binding site for xylanase GH11 [[6], PDB ID: 1TE1] is comprised of various segments of α-helices, α2 and α3 and loop α3-β4 (Figure 5). A simple docking analysis using discovery studio 2.0, insight II and O program [16,17] shows that Lys61, Lys98, Ser108 and Asn115 of XAIP-II form at least four hydrogen bonds with Glu179, Asn123 together with several van der Waals interactions. It may be noted that the main interacting segment of XAIP-II with xylanase GH11 is the loop α3-β4. The loop α3-β4 is considerably rigid in XAIP-II due to several intra loop interactions and hence lacks the freedom of getting induced fitting upon the binding of xylanase GH11. The corresponding loop in XAIP, on the other hand, is relatively flexible because it has only a few intra-loop interactions as a result it can be fitted well in the binding site of xylanase GH11 through induced fit. Thus the number of hydrogen bonded interactions between XAIP and xylanase GH11 are more than those between XAIP-II and xylanase GH11. Additionally, the side chain of His106 in XAIP forms two hydrogen bonds with Asp44 and Pro125 of xylanase (Figure 5A), while the corresponding Thr107 in XAIP-II does not form any hydrogen bond. Similarly, Asn98 in XAIP provides a superior hydrogen bond through Asn98 O^δ1 ^with Asn123 N^δ1^(Figure 5B) of xylanase than Lys98 of XAIP-II with Asn123 N^δ1^. The calculation of free energy [18] shows a higher value of -235 Kcal/mol for the interactions between XAIP and xylanase GH11 as compared to that of -195 Kcal/mol for XAIP-II and xylanase GH11. This is in full agreement with the dissociation constants of 4.5 × 10^-7 ^M for XAIP and 4.2 ×10^-6 ^M for XAIP-II which were determined using SPR method.

### Binding with α-amylase GH13

**Figure 6:**
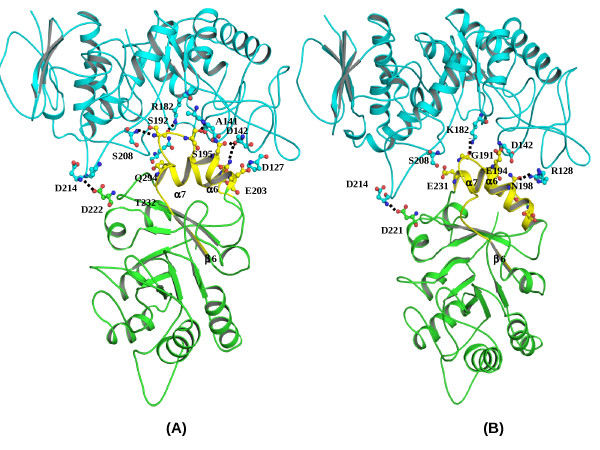
**The comparisons of interactions of α-amylase GH13 (blue) with (A) XAIP-II (green) and (B) XAIP (green)**. These were modeled using docking methods [16,17] are shown. The dotted lines indicate hydrogens bonds. The binding regions of both XAIP-II and XAIP are shown in yellow colour.

The binding site for α-amylase GH13 [[19], PDB ID: 1BLI] in XAIP-II is comprised of α-helices α6 (193-206) and α7 (230-243) and loops β6-α6 (180-192) and β7-α7 (218-230) (Figure 6). The most prominent difference is provided by the interaction of Ser192 in XAIP-II which is part of a tight type III' β-turn conformation. In contrast the corresponding tetrapeptide forms a type I' β-turn conformation with glycine residue at the equivalent site and lacks this important interaction. The molecular docking analysis carried out using discovery studio 2.0, insight II and O program [16,17] shows that there are seven hydrogen bonds and a number of van der Waals contacts at the interface between XAIP-II and α-amylase GH13. In comparison to this XAIP form only five hydrogen bonds and fewer van der Waals contacts. A free energy calculation [18] shows a higher value of interaction energy of -465 Kcal/mol between XAIP-II and α-amylase GH13 as compared to the value of -253 Kcal/mol for XAIP and α-amylase GH13. These values are also in agreement with the experimentally measured values of 3.4 × 10^-8 ^M and 3.6 × 10^-6 ^M of the dissociation constants for the dissociations of XAIP-II and XAIP respectively with α-amylase GH13.

## Discussion

The XAIP-II and XAIP are two forms of a protein that possess two independent interaction sites and inhibits two structurally unrelated enzymes xylanase GH11 and α- amylase GH13. Both xylanase GH11 and α-amylase GH13 hydrolyze plant related polymers xylan and starch respectively. Similarly, both XAIP-II and XAIP adopt stable TIM barrel folds. However, interestingly with only a few differences in their amino acid sequences on the common TIM barrel framework, the binding affinities of XAIP-II and XAIP varied significantly. XAIP-II binds to xylanase GH11 with an affinity lower than that of XAIP. The most important interactions of XAIP with xylanase GH11 are provided by His106 whereas the corresponding Thr107 in XAIP-II does not provide comparable interactions. The positioning of His106 in XAIP is a result of a unique conformation of the tripeptide Gly105-His106-Ser107 with two important intra tripeptide hydrogen bonds, Ser107 N --- O Gly105 = 3.0Ǻ and Ser107 O^γ ^--- O His106 = 2.7Ǻ. In contrast, the corresponding residue in XAIP-II is Thr107 which is positioned unfavorably due to an entirely different conformation of the corresponding tripeptide. The observed interactions clearly show that XAIP binds to xylanase GH11 more favourably than that of XAIP-II. On the other hand, XAIP-II inhibits the function of α-amylase GH13 with a considerable higher potency as shown by the dissociation constant of 3.4 × 10^-8 ^M whereas XAIP inhibits it with a lower potency as the dissociation constant in this case is 3.6 × 10^-6 ^M. In this regard, it is pertinent to note here that the amino acid changes between XAIP-II and XAIP have been observed primarily in the binding segments including loops α3-β4 and α4-β5 for the interactions with xylanase GH11 and in loops β6-α6 and β7-α7 and α-helices α6 and α7 for the interactions with α-amylase GH13. The insertion of an extra residue alanine at position 105 in the α3-β4 loop and replacements of His106 by Thr107 and Asn98 by Lys98 reduced both structural and chemical complementarities in XAIP-II with respect to the binding site in xylanase GH11 resulting in the formation of a less number of interactions with xylanase GH11. On the other hand the replacements of Gly191 by Ser192, Glu194 by Ser195, Glu231 by Ser232 and His236 by Glu237 made XAIP-II more compatible for the binding with α-amylase GH13 as compared to XAIP.

The existence of isoforms of enzymes is well known [20] and very often various organisms alter amino acid sequences in enzymes for altering their stereochemical arrangements for preventing the unwanted inhibitions of their functions. In this regard, the present example is one of the rare cases where the inhibitor proteins are found in more than one forms for protecting the host from the undesirable effects of hydrolytic enzymes presumably by alternating potencies so as to address the variations in the concentrations of enzymes Xylanase GH11 and α-amylase GH13.

## Conclusion

We have determined the structure of a new form of xylanase GH11 and α-amylase GH13 inhibitor protein (XAIP-II) at 1.2Ǻ resolution. The XAIP-II structure with slightly altered stereochemistry of its two independent binding sites shows that it binds to xylanase GH11 less favorably as compared to XAIP [1]. Whereas its α-amylase binding site shows stronger interactions as compared to those of XAIP. This specific interchange of inhibitory potencies demonstrates the potential of protein engineering by nature and allows us to have a deeper insight into the principle of selective replacements and insertions of amino acid residues in the proteins.

## Methods

### Purification of XAIP-II from *Scadoxus multiflorus*

The purification of XAIP-II was carried out using the modified procedure of Kumar et al. [1]. The samples of underground bulbs of *Scadoxus multiflorus *were collected from the nurseries located in the podhills of Himalayas. The bulbs were cut into small pieces and pulverized with the help of liquid nitrogen in a ventilated hood. The suspended material was dissolved in the extraction solution containing 0.2 M NaCl and 50 mM phosphate buffer, pH 7.2. An amount of 2.5 g of polyvinylpyrolidine (PVPP)/100 ml was added to the sample. The homogenate was centrifuged at 8000 g for 45 min at 4°C and the supernatant was collected. The ammonium sulphate was gradually added to the supernatant to make it to 80% saturation with constant stirring. This was incubated overnight on ice and centrifuged at 7000 g for 30 min. The pellet was removed and resuspended in the amount of 50 mM phosphate buffer, pH 7.2. It was dialyzed repeatedly against the same buffer with frequent changes for removing the salt. The dialyzed suspension was centrifuged at 8000 g for 30 min and the supernatant was loaded on DEAE-Sepharose A-50 column (50 × 2 cm) which was equilibrated with 50 mM phosphate buffer, pH 7.2. The protein was eluted using a linear gradient of 0.0 - 0.5 M NaCl in 50 mM phosphate buffer, pH 7.2. The second and third peaks in the elution profile were collected and pooled. These two peaks were identified with high and low inhibitions of xylanase (*Penicillium furniculosum*). The fraction that showed low inhibition of xylanase GH11 was collected and freeze-dried. This fraction was further gel filtered using Sephadex G-50 column (150 × 1 cm) with 50 mM phosphate buffer, pH 7.2 at a flow rate of 6 ml/hour. The first peak was collected, pooled and lyophilized. The sequence of the first 20 amino acid residues from N-terminus 1Gly-Ser-Leu-Asp-Ile-Ala-Val-Tyr-Trp-Gly-Gln-Ser-Phe-Asp-Glu-Arg-Ser-Asn-Glu-Ala20 was determined using automatic protein sequencer PPSQ21A (Shimadzu, Kyoto, Japan).

### Complete nucleotide sequence determination

In order to obtain the complete amino acid sequence of XAIP-II, the bulbs were sliced into small pieces and crushed into powder with Liquid Nitrogen. The total RNA was extracted using TRIZOL Reagent (Invitrogen, Carlsbad, USA). The cDNA synthesis was carried out from 10 ng of RNA with reverse transcription kit (Fermentas, Burlington, Canada) according to the manufacturer's instructions. The gene was amplified from the cDNA using a pair of primers. The forward primer 5'-GGCAGTCTGGACATCGCCGTC-3' derived from the N-terminal amino acid sequence of Gly-Ser-Leu-Asp-Ile-Ala-Val and the reverse primer 5'-CACGCCTTTGCCGAGGATCTT-3' obtained from the C-terminal amino acid sequence, Lys-Ile-Leu-Gly-Lys-Gly-Val were prepared. The PCR product was cloned in pGEMT-easy vector (Promega, Madison, USA) and the nucleotide sequence was obtained using ABI Prism 7000 (Applied Biosystem, Foster City, USA). The nucleotide sequence has been submitted in Genbank with an ID code of HM474410.

### Surface plasmon resonance studies of XAIP-II with xylanase GH11 and α-amylase GH13 enzymes

The method of surface plasmon resonance (SPR) was used for studying the binding properties of XAIP-II with xylanase GH11 [[6], PDB ID: 1TE1] and α-amylase GH13 [19, PDB ID: 1BLI]. All the SPR measurements were performed at 25°C using the BIAcore-2000 apparatus (Pharmacia Biosensor AB, Uppsala, Sweden) in which a biosensor-based system has been used for the real time specific interaction analysis. The sensor chip CM5, the amine coupling kit containing N-hydroxysuccinimide (NHS), N-ethyl-N'-3 (diethylaminopropyl) carbodiimide (EDC) and ethanolamine hydrochloride (Pharmacia Biosensor AB, Uppsala, Sweden) were used in the experiment. The running buffer used was 10 mM HBS-EP (pH 7.4) containing 0.005% surfactant P20. The sensor chip CM5 (disposable sensor chip, the surface of which was covered with a thin gold layer coated with carboxy-methyl dextran residue for covalent protein immobilization) was purchased from Pharmacia Biosensor AB (Uppsala, Sweden). The immobilization of XAIP-II was carried out at a flow rate of 10 μl/min at 25°C using amine coupling kit. The dextran on the chip was equilibrated with running buffer and carboxymethylated matrix was activated with an EDC/NHS mixture containing 210 μl of XAIP-II (80 μg/ml) in 10 mM sodium acetate (pH 4.6) was injected and unreacted groups were blocked by injecting ethanolamine. The SPR signal for immobilized XAIP-II was found to be 1254 RUs. Three different concentrations of the ligands, α-amylase and xylanase, 1.8 μM, 3.6 μM and 5.4 μM were prepared in 10 mM HBS-EP buffer (pH 7.4). These samples were then injected separately in two different flow cells, one with immobilized XAP-II and the other without XAIP-II as a reference to remove nonspecific binding with the surface of the chip in different cycles at a flow rate of 10 μl/min at 25°C. The dissociations of these ligands were induced by 10 mM HBS-EP buffer (pH 7.4). The rate constants K_A _and K_D _were obtained by fitting the primary sensorgram data using the BIA evaluation 3.0 software. The regeneration of the ligand bound to the surface of the protein was carried out using 0.1 mM NaOH. The kinetic parameters were obtained using the BIA evaluation software package. The association (k_on_) and dissociation (k_off_) rate constants for the ligand binding to XAIP-II were calculated and the value of the dissociation constant (KD) value was determined by the mass action relation K_D _= k_off _/k_on_.

### Crystallization of XAIP-II

The freshly purified samples of XAIP-II were dissolved in 20 mM phosphate buffer pH 7.2 to a final protein concentration of 20 mg/ml. The protein was crystallized with hanging drop vapour diffusion method at 293K using 24 well Limbro crystallization plates. The protein drops of 10 μl were equilibrated against the reservoir solution containing 0.1 M ammonium sulphate, 15 mM phosphate buffer, pH 7.2, 0.1 M sodium acetate and 10% (w/v) PEG 6000. The crystals grew to the maximum dimensions of 0.3 × 0.15 × 0.10 mm^3 ^within a period of three weeks.

### X-ray intensity data collection

The X-ray intensity data were collected using the DBT-sponsored synchrotron beamline ID 14-2 at the European Synchrotron Research Facility (ESRF) in Grenoble (France) using a MAR CCD detector (MAR USA Inc., Evanston, USA). In order to minimize the radiation damage, the crystal was placed in a nylone loop and kept at 100K in nitrogen stream during the measurements. The water ice formation was avoided by pre-incubation of the XAIP-II crystals for 3 minutes in the reservoir solution containing 22% (v/v) glycerol. The observed reflection data extended to a maximum resolution of 1.2Å. The reflection data were processed using DENZO and SCALEPACK from the HKL-2000 package [21]. Further data processing was carried out using programs from CCP4 package [22]. The crystals belong to monoclinic space group P2_1 _with unit cell dimensions of a = 42.2 b = 64.3, c = 48.6Å and β = 102.1° with one molecule in the asymmetric unit of the crystal unit cell. The crystal packing parameter Vm was calculated to be 2.2Å^3^/Da which corresponded to a solvent content of 44%. The data collection and processing statistics are summarized in Table 1.

**Table 1:** Data collection and refinement statistics

Space group	**P2**_**1**_
Unit cell dimensions	
a (Å)	42.2
b (Å)	64.3
c (Å)	48.6
β (°)	102.1
Number of molecules in the unit cell	2
Resolution range (Å)	36.0 - 1.2
The range of the highest shell	1.24 - 1.20
Total number of measured reflections	727277
Number of unique reflections	62459
Rsym (%)	5.0(22.1)
I/σ(I)	17.8(3.6)
Completeness of data (%)	98.5(87.1)
R_cryst _(%)	15.8
R_free _(%) 5% of reflections	18.6
Protein atoms	2101
Water oxygen atoms	434
Phosphate ion atoms (1)	5
Atoms from PEG	40
R.m.s.d in bond lengths (Å)	0.008
R.m.s.d in bond angles (°)	1.2
R.m.s.d in torsion angles (°)	16.8
**B-factors (Å^2^)**	
B-factor from Wilson plot (Å^2^)	12.3
Mean B-factor for main chain atoms (Å^2^)	11.2
Mean B-factor for side chain and water atoms (Å^2^)	19.2
Mean B-factor for all atoms(Å^2^)	15.8
**Ramachandran's ϕ, ψ map**	
Residues in the most favoured regions (%)	90.4
Residues in the additionally allowed regions (%)	9.3
Residues in the generously allowed regions (%)	0.3
	
PDB ID	3MU7

The numbers in the parentheses correspond to the data in the highest resolution shell

### Structure determination and refinement

The structure of XAIP-II was determined with molecular replacement method using coordinate of XAIP (PDB: 3D5H) as the search model. It was refined using the options of rigid body refinement, simulated annealing and energy minimization with program CNS [23] using data in the resolution range of 36.0 to 1.2Å. The conformations of loops were particularly examined by inspecting the composite OMIT maps using the programs REFMAC5 from CCP4 program suite [24] and COOT [25]. The refinement steps were repeated with intermitant manual building of the model. The positions of water oxygen atoms were determined manually using difference Fourier (|Fo - Fc|) maps on the basis of peak height and distance criteria. The water molecules whose thermal factors were 50Å^2 ^or above after refinement were removed from the list. Further model building and refinement cycles resulted in an R_cryst _of 0.158 and R_free _of 0.186 for 62,459 reflections from 36.0 to 1.2Å resolution. The average value of thermal B factor for all the atoms was 15.8Å^2^. The refinement statistics is given in Table 1.

## Abbreviations

DEAE: diethylaminoethyl cellulose; XAIP: xylanase - α-amylase inhibitor protein; TIM: triosephosphate isomerase.

## Authors' contributions

SK carried out the purification, crystallization, sequencing and structure analysis work. NS refined the structure, BM carried out SPR binding studies. DD performed modeling and docking work. MS carried out N-terminal sequencing and planned the complete sequence determination. SBS cloned, express and carried out nucleotide sequencing. SD planned and supervised binding studies. PK guided the molecular modeling and docking studies. SS planned the whole experiment, procured the material and supervised steps of various experiment. TPS designed experiments, analyzed the data and carried out detailes structural analysis and prepared the manuscript. All authors read and approved the final manuscript.
